# Unraveling the metabolic‒epigenetic nexus: a new frontier in cardiovascular disease treatment

**DOI:** 10.1038/s41419-025-07525-z

**Published:** 2025-03-18

**Authors:** Jun Ouyang, Deping Wu, Yumei Gan, Yuming Tang, Hui Wang, Jiangnan Huang

**Affiliations:** 1 Department of Cardiology, The First Affiliated Hospital of Guangxi Medical University, Nanning, Guangxi China; 2 Institute of Nephrology, Zhongda Hospital, Southeast University School of Medicine, Nanjing, Jiangsu China; 3 School of Pharmacy, Guangxi Medical University, Nanning, Guangxi China

**Keywords:** Cell biology, Post-translational modifications

## Abstract

Cardiovascular diseases are the leading causes of death worldwide. However, there are still shortcomings in the currently employed treatment methods for these diseases. Therefore, exploring the molecular mechanisms underlying cardiovascular diseases is an important avenue for developing new treatment strategies. Previous studies have confirmed that metabolic and epigenetic alterations are often involved in cardiovascular diseases across patients. Moreover, metabolic and epigenetic factors interact with each other and affect the progression of cardiovascular diseases in a coordinated manner. Lactylation is a novel posttranslational modification (PTM) that links metabolism with epigenetics and affects disease progression. Therefore, analyzing the crosstalk between cellular metabolic and epigenetic factors in cardiovascular diseases is expected to provide insights for the development of new treatment strategies. The purpose of this review is to describe the relationship between metabolic and epigenetic factors in heart development and cardiovascular diseases such as heart failure, myocardial infarction, and atherosclerosis, with a focus on acylation and methylation, and to propose potential therapeutic measures.

## Facts

1. Cardiovascular disease involves both metabolic and epigenetic alterations.
2. Metabolic and epigenetic factors interact with each other to synergistically promote cardiovascular disease progression.
3. The metabolic-epigenetic link is one of the determinants of cardiovascular disease.
4. At present, there is a lack of drugs that target both metabolic‒epigenetic links in cardiovascular disease treatment.

## Open questions

1. What metabolic and epigenetic alterations occur in cardiovascular disease?
2. How do metabolic and epigenetic factors interact with each other in the context of cardiovascular disease?
3. Can targeting metabolic‒epigenetic links lead to the development of new therapeutic drugs for cardiovascular diseases?

## Introduction

Cardiovascular disease is the most common cause of death worldwide and poses a considerable threat to people’s health [1]. Owing to the underlying complex pathological mechanisms, treatment methods for cardiovascular disease and its complications are still limited. Therefore, preventing and treating cardiovascular diseases is a long and arduous task for all medical workers. Studies have shown that cardiovascular diseases (atherosclerosis, myocardial infarction, heart failure, etc.) are often accompanied by alterations in cell metabolism [2–6]. Cellular metabolism provides basic energy for cellular activities, and intermediate metabolites also play essential roles in cellular activities. In recent years, research has shown that cellular metabolites can affect the epigenetic state of cells, leading to lasting gene expression changes and the formation of metabolic memory, which can still promote the development of cardiovascular diseases even after resolving the main triggers of the disease [7, 8]. During this process, cellular metabolites can directly serve as substrates for posttranslational modifications (PTMs) or indirectly affect the epigenetic status of cells by modulating the activity of epigenetic modifying enzymes [9]. Conversely, epigenetic alterations in cells can also affect the cellular metabolic status by influencing the gene expression of enzymes involved in glycolysis or the fatty acid oxidation pathway [10, 11]. The structural changes in chromatin during the above process constitute core pathological changes; therefore, chromatin is often regarded as a substrate for metabolic memory [7, 12, 13]. Furthermore, enzymes involved in metabolic pathways such as glycolysis and oxidative phosphorylation, can also undergo epigenetic modifications that affect their own activity and subsequently impact metabolic processes [14]. The persistent abnormal metabolic‒epigenetic cross-talk within cells plays a pivotal role in the progression of chronic diseases [7, 15]. Therefore, regulating the cellular metabolic‒epigenetic link may be the key to treating cardiovascular diseases. This review focuses on the relationship between cellular metabolic and epigenetic factors in diverse cardiovascular diseases, highlighting the importance of the cellular metabolic‒epigenetic link in cardiovascular diseases.

## Overview of the crosstalk between metabolic and epigenetic factors

### Diversity of metabolites and epigenetic factors

**Fig. 1:**
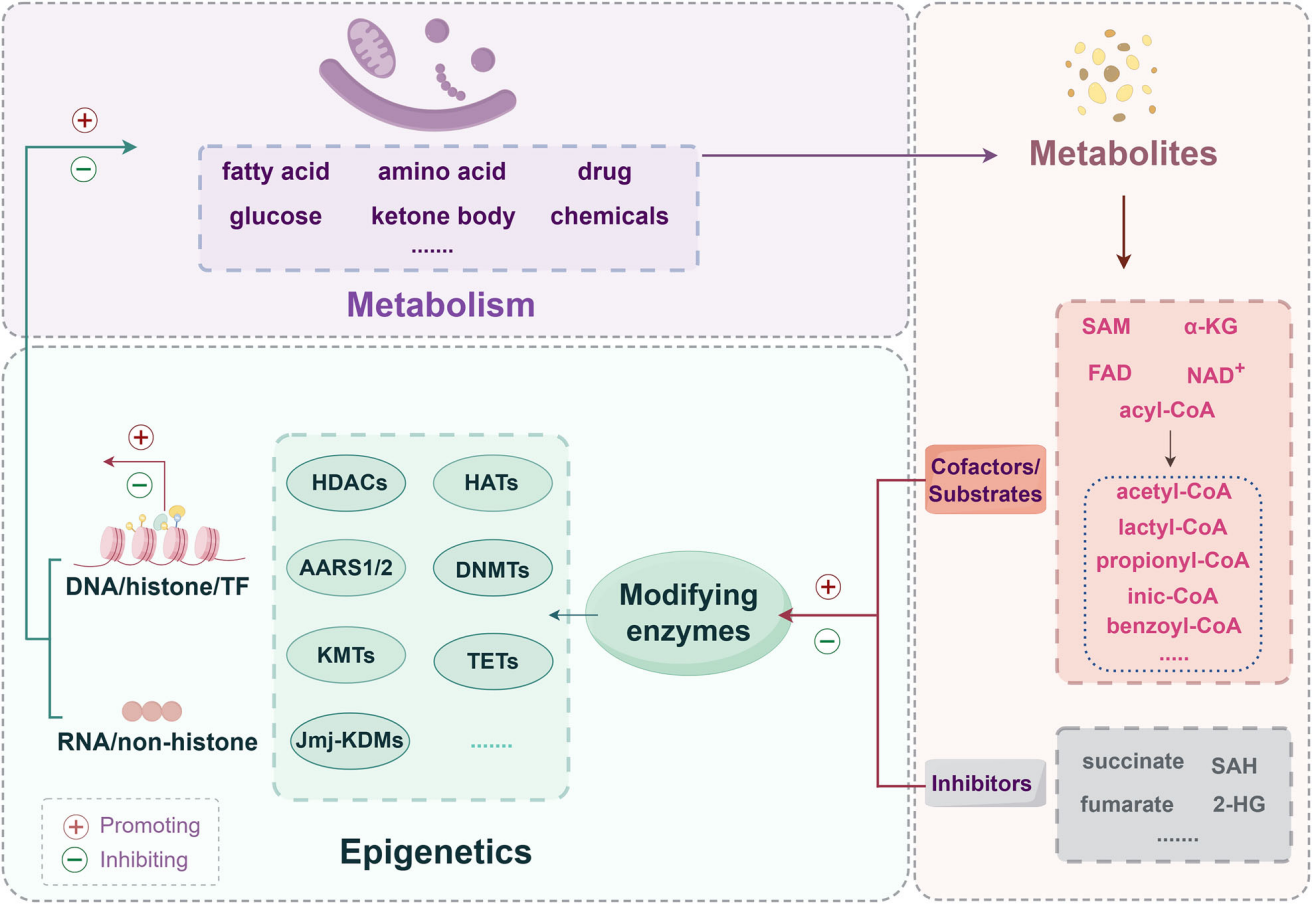
A brief diagram of the crosstalk between metabolic and epigenetic factors. Diverse metabolic processes such as fatty acid, amino acid, glucose, ketone body, drug, and chemicals metabolism can produce metabolite cofactors/substrates such as SAM, FAD, α-KG, and NAD^+^ and acyl-CoA or inhibitors such as succinate, SAH, fumarate, and 2-HG that can regulate the activity of epigenetic modifying enzymes such as HDACs, HATs, AARS1/2, DNMTs, KMTs, TETs, and Jmj-KDMs. The above metabolites can affect the epigenetic modifications of DNA, histones, and nonhistones in cells by acting on epigenetic modifying enzymes, ultimately affecting cellular metabolism or other cellular biological processes. SAM S-adenosylmethionine, FAD flavin adenine dinucleotide, α-KG α-ketoglutarate, NAD^+^ nicotinamide adenine dinucleotide, acyl-CoA acyl-coenzyme A, SAH S-adenosyl-homocysteine, 2-HG 2-hydroxyglutarate, HDACs histone deacetylases, HATs histone acetyltransferases, AARS1/2 alanyl-tRNA synthetase 1/2, DNMTs DNA methyltransferase, KMTs lysine methyltransferases, TETs ten-eleven translocation dioxygenases, Jmj-KDMs Jumonji lysine demethylases.

Metabolism and epigenetics are ubiquitous processes within cells that affect cell biology and play a role in pathological changes. In the past, it was generally thought that metabolic pathways merely provided energy for the cells and substrates required for biosynthesis to support cell growth and proliferation, whereas, epigenetics is an intricate process in which chemical modifications occur on nucleic acids and histone or nonhistone residues to alter chromatin structure or protein activity without altering genetic information and can even be transmitted to offspring through diverse mechanisms [16–18]. Many studies have confirmed that cellular metabolism plays a crucial role in various epigenetic changes and that changes in cellular metabolism ultimately affect the epigenetic state of cells, thereby widely influencing the biological or pathological behaviors of cells [7, 19–21] (Fig. 1). Therefore, cells can form a metabolic memory via epigenetics, and this memory may persist even after the metabolic disorder is corrected [22]. For example, in the process of hyperglycemia, diabetes may still occur even if blood glucose levels return to normal [23, 24].

Epigenetic modifying enzymes require metabolites from metabolic pathways to perform their modifying functions, thus establishing the link between metabolism and epigenetics [19, 25]. Common cellular metabolic processes include fatty acid metabolism, glucose metabolism, amino acid metabolism, and ketone body metabolism. The acetyl-CoA and S-adenosylmethionine (SAM) produced by the above pathways are important substrates for acetylation and methylation, respectively [26–31]. Additionally, nicotinamide adenine dinucleotide (NAD^+^) is a substrate for ADP-ribosylation [32, 33]. In addition to serving as substrates for epigenetic modifying enzymes, some metabolites can also act as cofactors for epigenetic modifying enzymes, such as α-ketoglutarate (α-KG), which is a cofactor for ten-eleven translocation (TET) dioxygenases and histone lysine demethylase (KDM) enzymes [8, 34–36]. Flavin adenine dinucleotide (FAD) is a cofactor of lysine-specific histone demethylase 1/2 (LSD1/2) [37–39]. With the development of high-performance liquid chromatography‒tandem mass spectrometry (HPLC‒MS/MS)technology, various new acylation modifications, such as propionylation, butyrylation, succinylation, malonylation, glutarylation, 2-hydroxyisobutyrylation, crotonylation, and β-hydroxybutyrylation induced by cognate fatty acids (propionate, butyrate, succinate, malonate, glutarate, 2-hydroxyisobutyrate, crotonate, and β-hydroxybutyrate) in the process of fatty acid metabolism [40, 41], have been continuously discovered (Fig. 1); additionally, a novel acylation modification (lactylation) induced by a metabolite (lactate) in the process of glycolysis has been reported recently [42, 43]. In addition, studies have shown that metabolites involved in drug metabolism (isoniazids) can also induce novel acylation modifications (isonicotinylation) [44, 45], and even metabolites involved in chemical additive metabolism (sodium benzoate) can also induce acylation modifications (benzoylation) [46–48]. The abovementioned novel acylation modifications involve a set of epigenetic modification enzymes with acetylation functions, namely, histone deacetylases (HDACs) and histone acetyltransferases (HATs). Thus, the influence of metabolites on epigenetics is so general. Undoubtedly, many new and unknown epigenetic modifications remain to be explored. On the other hand, unlike the above metabolites, which act as substrates or cofactors of epigenetic modifying enzymes, some metabolites can inhibit epigenetic modifying enzymes in direct or competitive manners. For example, succinate and fumarate are α-KG-dependent dioxygenase inhibitors [49, 50], whereas 2-hydroxyglutarate (2-HG), owing to its structural similarity to α-KG, can competitively inhibit α-KG-dependent dioxygenases [51, 52]. The metabolite S-adenosyl-homocysteine (SAH) from the methylation reaction is an inhibitor of methyltransferases [53, 54]. Therefore, a complex regulatory network is formed between metabolites and epigenetics in the body.

### Epigenetic modifying enzymes as mediators

Multiple epigenetic modifying enzymes in the body are responsible for linking metabolites with epigenetic modifications, thereby orchestrating cell biological and pathological processes (Fig. 1). These epigenetic modifying enzymes can be divided into two categories: those that promote modification, also known as “writers”, such as HATs and DNA methyltransferases (DNMTs), and those responsible for removing modification, also known as “erasers”, such as HDACs and TETs. HDACs and HATs are key enzymes that regulate the acylation of lysine residues [55, 56]. HDACs are composed of four subfamily members, namely, class I HDACs, class II HDACs (class IIa HDACs, class IIb HDACs), class III HDACs, and class IV HDACs. The class I HDACs are HDAC1-3 and HDAC8, which are distributed mainly in the nucleus and are involved in gene transcriptional repression [55, 57]. The class IIa HDACs are HDAC4-5, HDAC7, and HDAC9. The class IIb HDACs are HDAC6 and HDAC10. The class II HDACs are present mainly in the cytoplasm and can also be transported to the nucleus, which mainly plays a role in cellular signaling [55, 58]. Class III HDACs, also known as sirtuins (SIRTs), rely on NAD+ for deacylation. There are seven types of SIRTs in mammals: SIRT1-SIRT7. SIRT1-2 is present in both the nucleus and cytoplasm; SIRT3-5 is present in mitochondria; and SIRT6-7 is present only in the nucleus. The class IV HDACs include HDAC11, a unique member of the HDAC family that combines the characteristics of Class I and Class II HDACs and mainly participates in the immune response, inflammation regulation, and the occurrence of certain types of cancer [57, 59–61]. HATs are responsible for adding acyl groups to lysine residues to induce the acylation of target molecules. HATs are classified into two types on the basis of their cellular localization: nuclear type (Type A) and cytoplasmic type (Type B). Type A HATs include 5 families: (1) the P300/CBP family, which are the most famous HATs and are widely involved in cell signaling and gene transcription regulation [62, 63]; (2) the GNAT family, which typically works together with transcription activators and involves gene transcription [64]; (3) the MYST family (MOZ, YBF2/SAS3, SAS2, and TIP60 protein), which involves processes such as the cell cycle, DNA repair, gene transcription and cell death [65–68]; (4) the basal transcription factor (TF) family, which is essential for the initiation of transcription by RNA polymerase II (Pol II) [69, 70]; and (5) the NRCF (nuclear receptor cofactor) family, which interacts with nuclear receptors to regulate transcription in response to various physiological and environmental signals [71–74]. Type B HATs include HAT1, HAT2, Rtt109, HatB3.1, and HAT4 [75–77], which are responsible for regulating the acetylation of free histones in the cytoplasm. In addition to the above HATs, alanyl-tRNA synthetase 1/2 (AARS1/2) are novel lysine lactyltransferases for lactylation [78–80].

As a methyl group donor, SAM is an important substrate for methylation. Methylation can occur on various biological molecules, including DNA, RNA, proteins, and lipids. Methyltransferases are responsible for covalently binding methyl groups to target molecules, which is counteracted by demethylases. DNA methylation mainly occurs on the cytosine adjacent to a guanine (known as cytosine phosphate guanine (CpG)) and is catalyzed by DNMTs, including DNMT1, DNMT2, DNMT3A, and DNMT3b [81–83]. DNA methylation can inhibit or activate gene transcription, depending on the region and degree of methylation. Protein methylation can occur on various amino acid residues, including classic lysine, aspartic acid, arginine, histidine, and glycine residues by lysine methyltransferases (KMTs), aspartate methyltransferases, arginine methyltransferases, histidine methyltransferases, and glycine methyltransferases [84]. Acylation modification differs from these modifications as it mainly occurs on lysine residues. Furthermore, the degree of methylation is diverse and can be in mono-, di-, and trimethylation forms depending on certain conditions. Demethylases include ten-eleven translocation dioxygenases (TETs) and Jumonji lysine demethylases (Jmj-KDMs) [85–88]. Therefore, the regulation of intracellular methylation is much more intricate than the regulation of acylation. Notably, epigenetic modifying enzymes that regulate acetylation and methylation are not specific to histones or DNA, and other nonhistone proteins and RNA can also undergo epigenetic modifications. Taken together, the above epigenetic modifying enzymes closely link intracellular metabolic processes with epigenetics by adding or removing metabolite acyl/methyl groups to or from target molecule residues (Fig. 1).

## Heart development

The heart is the first functional organ to develop in the embryo [89]; it gradually forms four chambers during its development and is composed of structures such as the cardiac conduction system, myocardial inner and outer membranes, and heart valves [90]. These structures consist of multiple cell types originating from two pools of embryonic field progenitor cells [91]. The first heart field (FHF) progenitor cells mainly give rise to cardiomyocytes and form the left ventricle (LV) and part of the atrium [92, 93], whereas the second heart field (SHF) progenitor cells perform versatile functions. In addition to forming cardiomyocytes, they can also produce cardiac endothelial cells, smooth muscle cells, outflow and inflow channels, as well as cardiomyocytes in the conduction system, and form the right ventricle, outflow channel, and atrium [94–98]. The abovementioned myocardial cells and nonmyocardial cells coordinate and precisely regulate each other to ultimately form a whole heart [99]. Any problem at any stage of the development of these cell types could result in embryonic lethality or congenital heart disease (CHD).

In the past, it was commonly believed that the development of cardiac cells was merely influenced by genetic processes [100]. However, epigenetic modifications, especially methylation and acetylation, have been confirmed to play crucial roles in regulating the development of cardiac cells [89, 101]. Acetylation and methylation require metabolites such as acetyl and methyl groups as substrates. Therefore, the metabolic status of cardiac cells can affect their methylation and acetylation, thereby affecting their growth and development.

To date, most studies have investigated the role of epigenetics in heart development by knocking out epigenetic modifying enzymes. Research has shown that HDACs play crucial roles in cardiac development and CHD [102]. The knockout of both HDAC1 and HDAC2 in the heart can lead to severe arrhythmia and dilated cardiomyopathy, whereas the deletion of one gene does not affect heart development [103], indicating functional redundancy between HDAC1 and HDAC2. HDAC5 and HDAC9 exhibit similar behaviors to those of HDAC1 and HDAC2 [102]. Double knockout of HDAC5 and HDAC9 in the heart can lead to fatal ventricular septal defects (VSDs) and thin-walled myocardium, whereas single knockout does not affect cardiac development [102, 104]. HDAC3 is also essential for cardiac development, and knocking out HDAC3 can lead to various cardiac developmental defects and embryonic lethality [105]. In addition, HDAC3 is involved in myocardial metabolism. Knocking out HDAC3 promotes oxidative phosphorylation in myocardial cells while inhibiting glucose metabolism [105]. It is currently unclear whether knocking out HDAC3 inhibits cardiac development by affecting myocardial metabolism. Cardiac development was not significantly affected by HDAC8 or HDAC11 knockdown alone. Additionally, class III HDACs (SIRTs) play pivotal roles in cardiac hypertrophy and disease, as detailed in other excellent reviews [106–108]. In addition to HDACs, HATs also play crucial roles in cardiac development, with P300 being the most extensively studied [109]. Homozygous p300 knockout mice exhibit defects in cardiac development, reduced formation of cardiac trabeculae, and decreased expression of cardiac structural proteins such as α-MHC and α-actin [110, 111]. However, the overexpression of cardiac P300 can also lead to abnormal cardiac development, indicating that the activity and expression of P300 need to be maintained at appropriate levels during cardiac development [112–114]. Furthermore, DNA and histone methylations are present throughout the entire stages of cardiac development and maturation and remain dynamic [115]. The role of methylation in cardiac development is more complex than that of acetylation because of the diversity of modifying enzymes involved in methylation and their substrates. There have been many excellent reviews detailing the critical role of methylation in cardiac development [116–119].

**Fig. 2:**
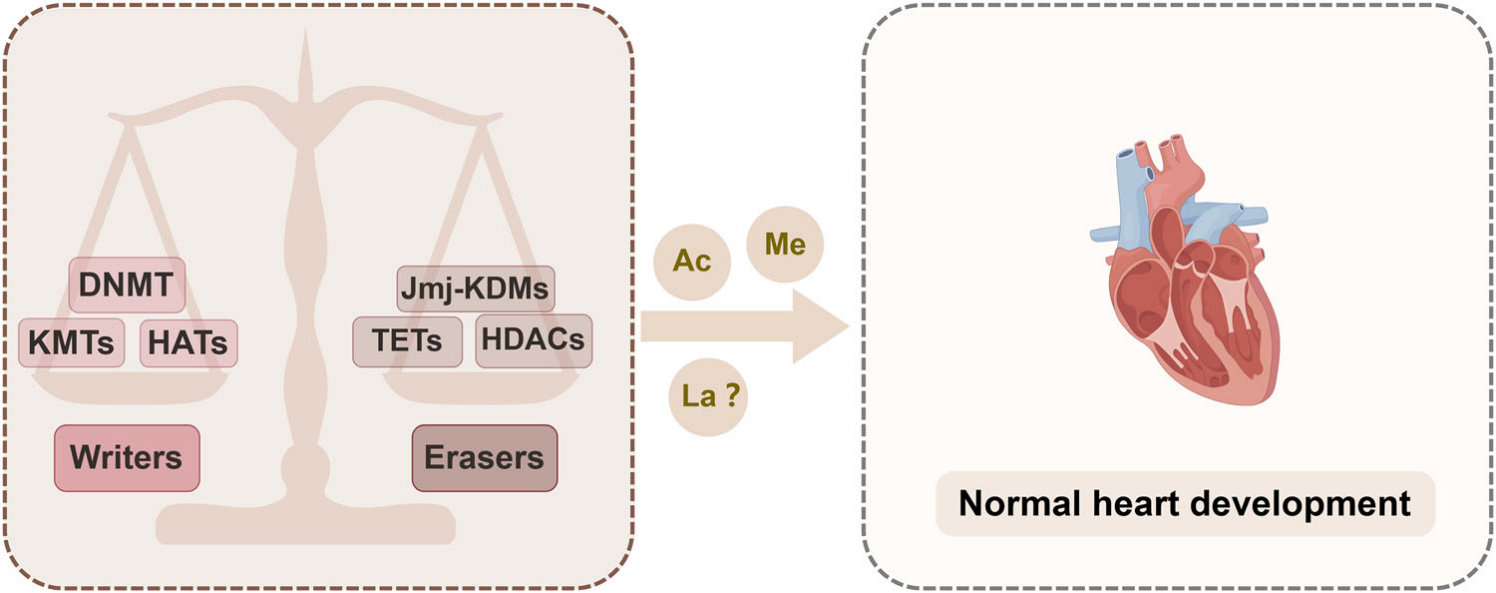
The importance of balance among epigenetic modifying enzymes in heart development. “Writer” proteins such as DNMTs, KMTs, and HATs, as well as “Eraser” proteins such as Jmj-KDMs, TETs, and HDACs, coordinately maintain cellular epigenetic homeostasis through metabolites such as acetyl-CoA, methyl, and perhaps lactyl-CoA, which is crucial for cardiac development. DNMTs DNA methyltransferase, KMTs lysine methyltransferases, HATs histone acetyltransferases, Jmj-KDMs Jumonji lysine demethylases, TETs ten-eleven translocation dioxygenases, HDACs histone deacetylases, acetyl-CoA acetyl-coenzyme A, lactyl-CoA lactyl-coenzyme A.

During the embryonic stage, the heart relies mainly on glycolysis for energy production; however, after birth, fatty acid oxidation gradually becomes the main pathway for energy production [120]. Recent studies have shown that the final metabolic product of glycolysis, lactate, can induce a novel epigenetic modification, lactylation [42]. Previous studies have reported that lactate is involved in the development of the mouse brain [121, 122]. However, whether lactate can participate in heart development by inducing protein lactylation in the fetal stage remains to be explored. In general, epigenetic modifying enzymes can utilize cellular metabolites such as methyl, acetyl, and other possible acyl groups to regulate DNA or protein modifications, thereby affecting heart development. Therefore, adequate raw materials and a balance among epigenetic modifying enzyme activity or expression are prerequisites for ensuring normal heart development (Fig. 2).

## Heart failure

Heart failure is a clinical syndrome characterized by a severe decline in cardiac function caused by various etiologies [123]. More than 50% of heart failure patients experience heart failure with a preserved ejection fraction [124]. Research has shown that, regardless of etiology, heart failure is associated with metabolic disorders and abnormal gene expression [7]. Abnormal metabolism in the heart promotes the occurrence of heart failure by regulating histone, nonhistone, and DNA epigenetic modifications [125–128].

In healthy hearts, cardiomyocyte metabolism is flexible [129]. Adult cardiomyocytes mainly rely on fatty acid β-oxidation for energy production [130]. Compared with fatty acid β-oxidation, glycolysis is the more efficient pathway in terms of energy supply [131]. During prenatal cardiac stress, the rate of glycolysis in cardiomyocytes increases [132]. Lactate is also an important energy substrate of the heart and a critical signaling molecule [133–137]. Studies have shown that lactate may be an important source of pyruvate in the heart [138]. During periods of starvation, ketone bodies produced by the liver can also serve as a source of energy for the heart [129, 139–142]. In addition, cardiomyocytes can also be powered by branched chain amino acids (BCAAs) [143]. Therefore, healthy cardiomyocytes can obtain energy through various pathways mentioned above.

**Fig. 3:**
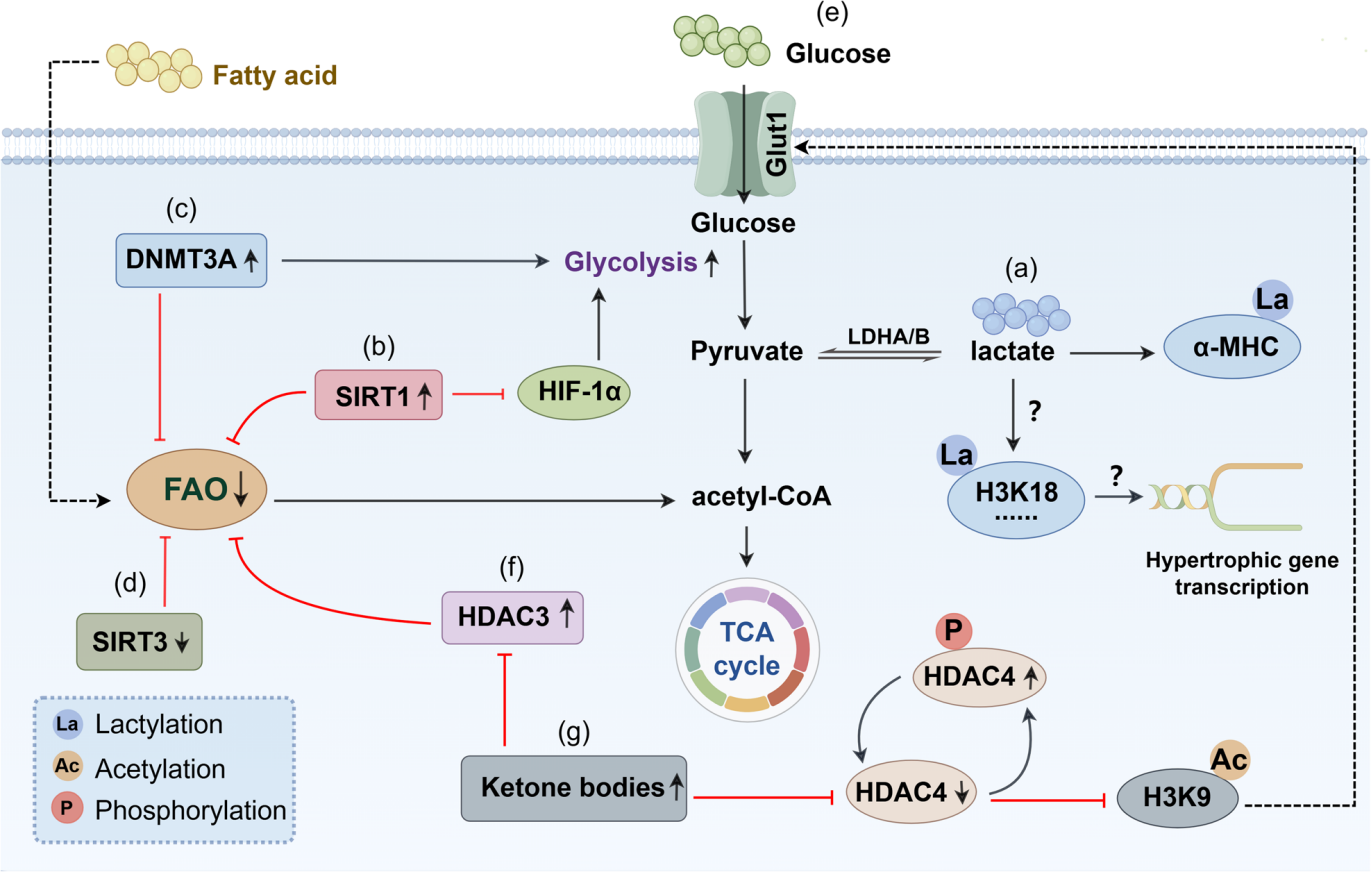
Crosstalk between metabolic and epigenetic factors in heart failure. **a** The rate of glycolysis increases in cardiomyocytes, and the metabolic end product lactate can induce α-MHC lactylation and H3K18la, thereby affecting protein function or gene expression in the failing heart. **b** The expression level of SIRT1 increases, which can inhibit glycolysis and fatty acid oxidation during heart failure. **c** The expression level of DNMT3A increases, which can promote glycolysis and inhibit fatty acid oxidation during heart failure. **d** The expression level of SIRT3 decreases, which can inhibit fatty acid oxidation during heart failure. **e** Exercise can lower glucose levels, thereby increasing glucose metabolism and improving heart function. **f** The expression level of HDAC3 increases, which can inhibit fatty acid oxidation in the failing heart. **g** Ketone bodies can inhibit HDACs such as HDAC3 and HDAC4 in cardiomyocytes, thereby improving cardiac function in heart failure. α-MHC α- myosin heavy chain, HIF-1α hypoxia inducible factor, FAO fatty acid β-oxidation, HDAC3 histone deacetylase 3, HDAC4 histone deacetylase 4, DNMT3A DNA methyltransferase 3A, Glut1 glucose transporter 1, LDHA/B lactate dehydrogenase A/B.

However, in the failing heart, metabolism is inflexible [144, 145]. Failing heart is characterized by decreased fatty acid oxidation, increased glycolysis, and an increased reliance on ketone body oxidation [146–149]. Metabolic changes in the failing heart ultimately lead to changes in the composition and content of intracellular metabolites, as well as epigenetic alterations (Fig. 3). For example, ketone bodies can protect the failing heart. Multiple mechanisms involve the cardioprotective effects of ketone bodies: (1) providing auxiliary fuel for the heart; (2) modulating the utilization of fatty acids and glucose by cardiomyocytes; and (3) mitigating cardiac oxidative stress and cardiac hypertrophy by inhibiting the epigenetic modifying enzymes HDACs [150, 151], which is consistent with previous findings that the inhibition of HDACs can alleviate cardiac hypertrophy and remodeling in heart failure [152–154]. Moreover, recent studies have shown that ketone bodies can protect myocardial mitochondria by abrogating the transcriptional repression of the peroxisome proliferator-activated receptor-γ coactivator 1α (PGC1α) gene by H3K27me2K36me1 [155]. Therefore, administering an appropriate amount of ketone bodies may ameliorate heart failure. In addition to the epigenetic effects of ketone bodies and glycolysis end products, lactate can induce epigenetic modification–lactylation [42, 156]. Research has shown that a reduction in the level of α-myosin heavy chain (α-MHC) K1897 lactylation in cardiomyocytes can hinder the interaction between α-MHC and Titin, thereby damaging the structure and reducing the function of the heart and exacerbating heart failure [157]. Conversely, increasing the level of α-MHC K1897 lactylation by increasing the lactate concentration in cardiomyocytes can attenuate heart failure [157]. However, a recent study on cardiac hypertrophy reported that an increase in H3K18la levels in cardiomyocytes promoted cardiac hypertrophy, whereas reducing H3K18la levels by inhibiting lactate levels in cardiomyocytes significantly alleviated cardiac hypertrophy [127]. It is unclear whether cardiomyocytes in heart failure also exhibit lactylation at H3K18 or other important sites.

On the other hand, studies have shown that epigenetic modifying enzymes can also affect the energy metabolism of cardiomyocytes in heart failure. For example, DNMTs, especially DNMT3A, which is highly expressed in cardiomyocytes, are associated with mitochondrial structure, function, and lipid metabolism [158]. DNMT3A hypermethylation promotes glycolysis in cardiomyocytes while inhibiting fatty acid oxidation in the failing heart [159]. In addition to methylation, acetylation is closely related to myocardial energy metabolism in the failing heart [160, 161]. Acetylated proteins are enzymes involved in glycolysis, glucose oxidation, fatty acid β-oxidation, the electron transport chain, and the tricarboxylic acid cycle [162, 163]. For example, SIRT1 can inhibit glycolysis through SIRT1-mediated hypoxia inducible factor (HIF-1α) deacetylation [164]. In addition, SIRT1 can inhibit fatty acid β oxidation in failing hearts by suppressing estrogen-related receptor (ERR) target genes and heterodimerizing peroxisome proliferator-activated receptor (PPARα) and retinoid X receptor α (RXRα) [165, 166]. SIRT3 also plays an important role in heart failure. One of the potential cardioprotective mechanisms of SIRT3 is to regulate the deacetylation of enzymes involved in mitochondrial metabolism, including long-chain acyl CoA dehydrogenase (LCAD) and β-hydroxyacyl CoA dehydrogenase (β-HAD) in fatty acid β-oxidation, pyruvate dehydrogenase (PDH) in glucose oxidation, and enzymes of the tricarboxylic acid cycle and the electron transport chain, while hyperacetylation of the aforementioned enzymes can impair cardiac energy production in heart failure [162, 167–175].

In summary, during heart failure, the heart undergoes a series of complex metabolic and epigenetic alterations. Metabolites such as ketone bodies and lactate participate in the occurrence of heart failure by inhibiting epigenetic modifying enzymes or directly inducing lactylation. On the other hand, various epigenetic modifying enzymes can also directly or indirectly affect cardiac metabolism through multiple mechanisms (Fig. 3).

## Myocardial ischemia/reperfusion injury (MIRI)

Myocardial infarction (MI) is one of the leading causes of death worldwide and is caused by a sharp decrease in or cessation of blood flow to cardiomyocytes [176, 177]. Timely restoration of the coronary artery blood flow supply is a routine treatment for MI [178, 179]. However, reperfusion therapy can cause MIRI, further exacerbating myocardial injury [180–182]. The pathophysiological mechanisms of MIRI are very complex and include oxidative stress, the inflammatory response, mitochondrial dysfunction, apoptosis, and autophagy [183], but the specific mechanisms are still not well-defined.

**Fig. 4:**
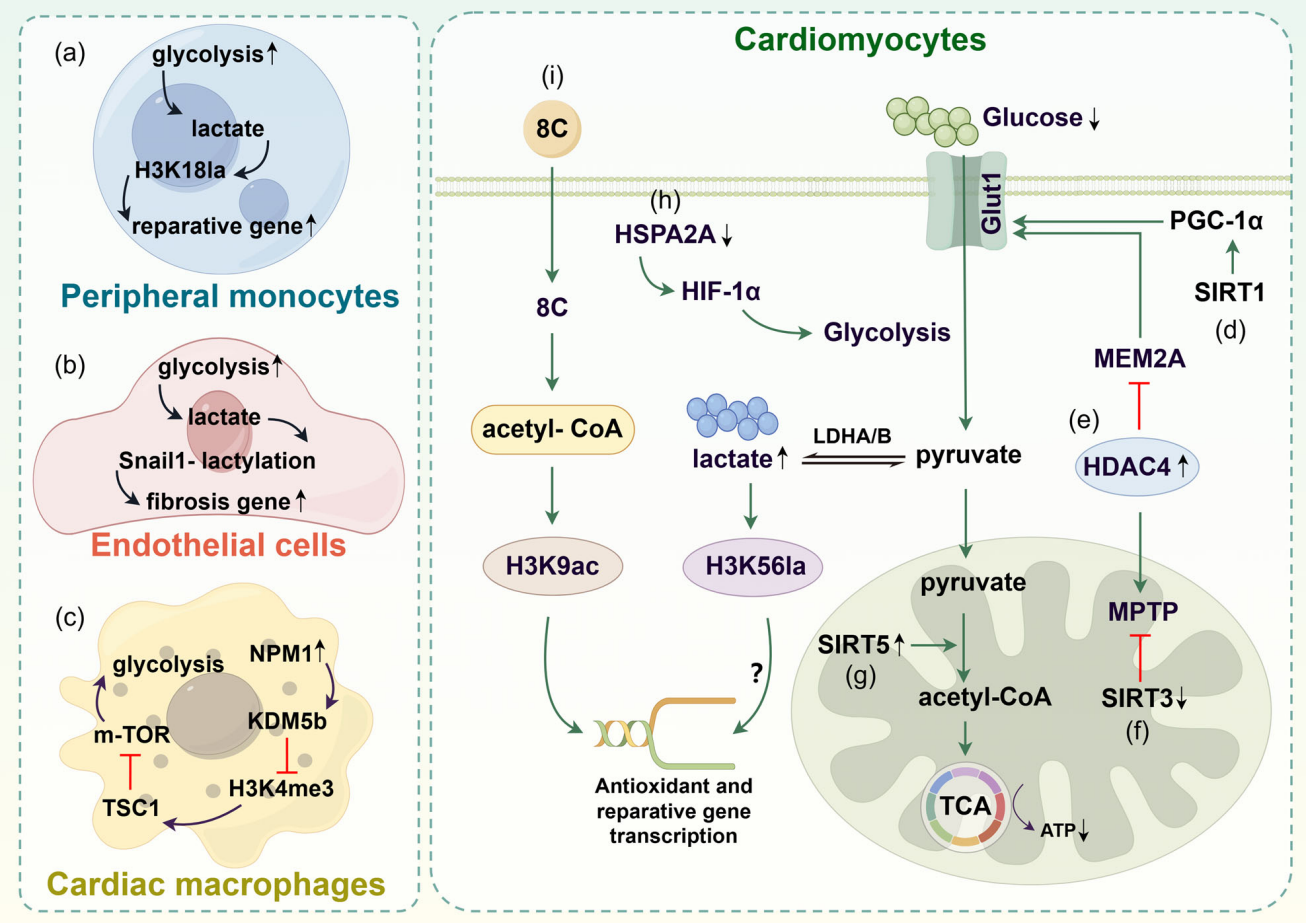
Crosstalk between metabolic and epigenetic factors in MIRI. **a** In the early stage of MI, the rate of glycolysis in peripheral monocytes increases, and the end product lactate can induce H3K18la and drive the expression of reparative genes. **b** In post-MI, the rate of glycolysis in endothelial cells increases, and lactate-induced Snail1 lactylation can drive the expression of fibrotic genes. **c** In MI, the level of NPM1 in cardiac macrophages increases, which can promote glycolysis through H3K4me3 on the TSC1 promoter. **d** SIRT1 promotes glucose metabolism in cardiomyocytes by regulating PGC1-α. **e** The expression level of HDAC4 increases in cardiomyocytes, which can inhibit glucose metabolism by inhibiting MEM2A and promoting MPTP opening. **f** The expression level of SIRT3 decreases in cardiomyocytes, which can promote MPTP opening. **g** The expression level of SIRT5 increases in cardiomyocytes, which can promote the conversion of pyruvate to acetyl-CoA. **h** In MIRI, HSPA2A can promote H3K56la by increasing the glycolysis rate in cardiomyocytes. **i** In MIRI, 8C-induced H3K9ac in cardiomyocytes can increase the expression of reparative genes. MI Myocardial infarction, NPM1 nucleophosphmin 1, TSC1 TSC complex subunit 1, lysine demethylases 5b (KDM5b) m-TOR mammalian target of rapamycin, HDAC4 histone deacetylase 4, MEM2A myocyte enhancer factor 2A, MPTP mitochondrial permeability transition pore, acetyl-CoA acetyl-coenzyme A, MIRI myocardial ischemia‒reperfusion injury, HSPA2A heat shock protein A12A, 8C sodium octanoate, PGC1α peroxisome proliferator-activated receptor-γ coactivator 1α, HIF-1α hypoxia inducible factor, Glut1 glucose transporter 1, LDHA/B lactate dehydrogenase A/B.

Research has shown that both MI patients and MIRI patients exhibit abnormal energy metabolism and epigenetic alterations in specific cells [184, 185] (Fig. 4). Modulating metabolic or epigenetic status has great value in the treatment of MI or MIRI. Under ischemic conditions, mitochondrial oxidative phosphorylation in cardiomyocytes decreases [3]. To meet the energy supply of cardiomyocytes, the rate of anaerobic glycolysis increases [186]. However, this metabolic transition greatly reduces the energy supply of cardiomyocytes. In the absence of reperfusion, glucose is mainly produced in cardiomyocytes by the breakdown of glycogen stored within the cells [3]. During anaerobic glycolysis, glucose is continuously consumed. However, the acidosis caused by lactate accumulation inhibits enzymes involved in glycolysis, ultimately leading to the complete termination of glycolysis [3]. Persistent ATP deficiency and cellular acidosis cause irreversible damage to cardiomyocytes. However, research has shown that aerobic glycolysis has a protective effect on cardiomyocytes in MIRI [187]. A previous study reported that in the early stage of MIRI, the level of heat shock protein A12A (HSPA12A), an atypical member of the HSP70 family, was decreased in cardiomyocytes. Overexpression of HSPA12A can protect cardiomyocytes by increasing the stability of the Hif-1α protein in a smerf1-dependent manner, which can promote aerobic glycolysis while maintaining lactate-induced lactylation of H3 at lysine 56 (H3K56la) [188]. Unfortunately, the specific molecular mechanism by which H3K56la participates in protecting cardiomyocytes is not clear in MIRI; perhaps it regulates the transcription of cardiac repair genes to achieve cardiomyocyte protection. In addition, in the early-stage post-MI, the rate of glycolysis increases in peripheral monocytes. The metabolite lactate can induce H3K18la in monocytes to regulate the expression of repair genes and their polarization into reparative macrophages [189]. Reparative macrophages are recruited to the myocardial injury area to repair the injured tissue [189]. However, glycolysis can impair the repair function of cardiac macrophages. A recent study revealed that in MI, NPM1 (nucleophosphmin1) in cardiac macrophages promotes inflammatory glycolysis by recruiting KDM5b to inhibit the trimethylation of histone H3 at lysine 4 (H3K4me3) on the TSC1 promoter and inhibit its transcription [190]. Additionally, Snail1 lactylation promotes endothelial-to-mesenchymal transition (EndMT), which exacerbates cardiac fibrosis post-MI [191]. In addition to the metabolite lactate, which can play a role in epigenetics, acetyl-CoA generated from the medium-chain fatty acid 8 C can induce the acetylation of histone H3 at lysine 9 (H3K9ac) through the histone acetyltransferase Kat2a in MI, thereby promoting cardiomyocyte antioxidant and reparative gene transcription [192].

Multiple studies have shown that HDACs also play crucial roles in myocardial energy metabolism during MI or MIRI [160, 184, 185, 193, 194]. Research has shown that in MI, HDAC4 reduces GLUT1 expression by inhibiting MEM2A, impairing glucose metabolism [195]. In addition, HDAC4 can also increase the opening of mitochondrial permeability transition pores (mPTPs) and mitochondrial apoptosis, leading to mitochondrial dysfunction [160]. Additionally, during MIRI, SIRT1 promotes glucose metabolism in cardiomyocytes by regulating PGC1-α [196], while SIRT3 can regulate mitochondrial biogenesis and inhibit the opening of mPTPs [197], and SIRT5 can accelerate the conversion of pyruvate to acetyl-CoA, improving cardiac function [198]. However, there is limited research on HATs and SIRT2, 4, 6, 7 in myocardial energy metabolism during MIRI.

In addition to acetylation, DNA methylation also plays an important role in MI. In perinatal neonatal rats exposed to nicotine, increased levels of DNA methylation and DNMT3 can aggravate MIRI [199]. However, it is currently unclear whether methylation participates in MIRI by affecting myocardial energy metabolism. Overall, in MI or MIRI, cardiomyocytes or macrophages feature remarkable metabolic and epigenetic alterations, which interact with each other and synergistically affect the occurrence and development of diseases.

## Atherosclerosis

**Fig. 5:**
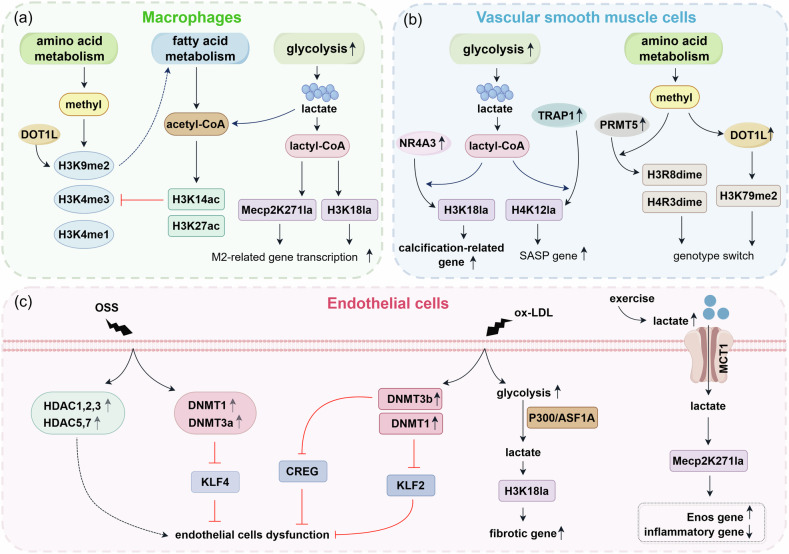
Crosstalk between metabolic and epigenetic factors in atherosclerosis. **a** In atherosclerosis, methyl, acetyl-CoA, and lactyl-CoA are produced during amino acid metabolism, fatty acid metabolism, and glycolysis, respectively. Methyl can induce H3K9me2, H3K4me3, and H3K4me1 in macrophages, while acetyl-CoA can induce H3K27ac. In addition, DOT1L in macrophages can promote lipid metabolism by inducing H3K9me2. Acetyl-CoA may inhibit H3K4me3 by inducing H3K14ac. Lactyl-CoA can induce Mecp2K271la and H3K18la, promoting the expression of M2 macrophage-related genes. **b** In atherosclerosis, the rate of glycolysis in VSMCs increases, and the end product lactate can induce H3K18la and H4K12la under NR4A3 and TRAP1, respectively, thus promoting the expression of calcification-related genes and SASP-related genes, respectively. In addition, the levels of PRMT5 and DOT1L are increased in VSMCs, while the levels of the H3R8 dimer, H4R3 dimer, and H3K79me are increased, thereby mediating phenotype switching. **c** Under oscillatory shear stress stimulation, the levels of HDAC1, 2, 3, 5, and 7; DNMT1; and DNMT3a increase in endothelial cells, leading to endothelial cell dysfunction. Under ox-LDL stimulation, the levels of DNMT1 and DNMT3b in endothelial cells increase, leading to endothelial cell dysfunction. Moreover, the rate of glycolysis in endothelial cells increases and induces H3K18la, promoting fibrotic gene expression. In addition, exercise can lead to an increase in the level of lactate in the body, which enters endothelial cells through MCT1 on the cell surface and induces Mecp2K271la, promoting Enos gene expression and inhibiting proinflammatory gene expression. acetyl-CoA acetyl-coenzyme A, lactyl-CoA lactyl-coenzyme A, DOT1L disruptor of telomeric silencing-1-like, Mecp2K271la methyl-CpG binding protein 2 (MeCP2) K271 lactylation, NR4A3 Nuclear receptor 4A3, TRAP1 Tumor neurois factor receptor associated protein 1, SASP senescence-associated secretory phenotype, PRMT5 protein arginine methyltransferase 5, VSMCs Vascular smooth muscle cells, OSS oscillatory shear stress, HDAC1,2,3,5,7 Histone deacetylase 1,2,3,5,7, ECs Endothelial cells, ox-LDL oxidized low-density lipoprotein, DNMT1 DNA methyltransferase 1, DNMT3a DNA methyltransferase 3a, DNMT3b DNA methyltransferase 3b, MCT1 Monocarboxylate transporter 1, Kruppel-like factor 2/4 KLF 2/4, CREG cellular repressor of E1A-stimulated gene, ASF1A Anti-silencing factor 1A.

Atherosclerosis is a chronic low-grade inflammatory disease caused by lipoprotein deposition and the subsequent immune response, and it is also an important cause of cardio-cerebrovascular diseases such as stroke, coronary heart disease, and peripheral vascular disease [200–205]. In addition, atherosclerosis is a disease involving multiple types of cells, including macrophages, endothelial cells, vascular smooth muscle cells (VSMCs), and lymphocytes, of which macrophages are the most abundant immune cell type in atherosclerotic plaque tissue [206, 207]. The pathological changes in these cells are crucial triggers of atherosclerosis. Studies have confirmed that in addition to genetic impact, metabolic and epigenetic factors are also involved in the pathological changes in cells during atherosclerosis (Fig. 5). Numerous studies have shown that atherosclerosis is characterized by trained immunity [208, 209]. Trained immunity involves immune cells, such as macrophages, and nonimmune cells, such as endothelial cells and VSMCs [210]. Trained immunity refers to a rapid response with increased strength to secondary stimuli similar to or completely unrelated to the initial stimulus, which is constructed via the crosstalk between metabolic and epigenetic factors [206, 211]. The molecular basis is the chromatin structural changes and other epigenetic alterations induced by metabolites, which can persist after the first stimulus is released [212].

### Macrophages

Macrophages are known to be highly plastic. On the basis of their function and surface markers, macrophages are classified into diverse subtypes in atherosclerotic plaque tissue, including proinflammatory macrophages (M1), anti-inflammatory macrophages (M2a, M2b, and M2c), Mox, M4, M (Hb), and Mhem macrophages [206]. Among them, M1 and M2 macrophages account for the largest proportion (40% and 20%, respectively) and are also the most extensively studied [213]. During atherosclerosis, macrophages undergo metabolic and epigenetic changes (Fig. 5). Both the presence of oxidized low-density lipoprotein (ox-LDL) and hypoxic stimulation increase HIF-1a expression in macrophages, which in turn promotes glycolysis and lactate accumulation [206]. Recent studies have shown that lactate in macrophages plays an important role in atherosclerosis. H3K18la induced by lactate can promote the polarization of M2 macrophages, thereby inhibiting atherosclerosis [214]. In addition, lactate can induce methyl-CpG binding protein 2 lactylation at K271 (Mecp2K271la), which can regulate the expression of reparative macrophage-associated genes, ultimately stabilizing plaques and reducing the risk of atherosclerotic cardiovascular disease [215].

Macrophages also undergo methylation and acetylation during atherosclerosis, such as H3K9me2, H3K4me3, H3K4me1, and H3K27ac [216–218]. After stimulation was removed, H3K27ac in macrophages disappeared, whereas H3K4me3 and H3K4me1 remained [219], indicating that H3K4me3 and H3K4me1 may serve as epigenetic memories in macrophages, whereas H3K27ac acts as a promoter activity marker [220]. Furthermore, studies have shown that inhibiting glycolysis can suppress H3K4me3 modification and trained immunity in macrophages. However, the specific mechanism by which glycolysis inhibits trained immunity in macrophages is not clear. The possible mechanism is that acetyl-CoA produced by glycolysis can stimulate H3K14ac modification [221], which can promote H3K4me3 modification [222]. Furthermore, a recent study revealed that H3K79me2, which is mediated by the disruptor of telomeric silencing-1-like (DOT1L), can promote lipid metabolism in macrophages, thereby inhibiting inflammatory responses [223]. The role of DNA methylation in the trained immunity of macrophages in the context of atherosclerosis has not been well studied and needs further exploration.

### Vascular smooth muscle cells

Vascular smooth muscle cells (VSMCs) are the main cellular component of the middle layer of the blood vessel wall [224]. Like macrophages, VSMCs also exhibit significant plasticity and can change between contractile and secretory (or synthetic) types [225–227]. Osteochondrogenic, premature senescent, mesenchymal stem cell-like, macrophage-like, adipogenic, inflammatory, and aberrantly proliferative and migrated VSMCs are pathological phenotypes [227, 228]. VSMCs participate in atherosclerosis mainly via various pathological phenotypes. However, at present, the molecular mechanism regulating the pathological phenotypes of VSMCs in the context of atherosclerosis is not well established. Studies have shown that VSMCs also undergo significant metabolic and epigenetic changes during atherosclerosis (Fig. 5). In the process of atherosclerosis, VSMCs change from the contractile type to the synthetic type, which is usually accompanied by increased aerobic glycolysis [229]. The glycolytic metabolite lactate can regulate the epigenetic status of VSMCs by mediating lactylation. For example, nuclear receptor 4A3 (NR4A3) can promote lactate-induced H3K18la modification in calcifying VSMCs, which can promote the expression of Phospho1 (phosphotase orphan1), aggravating vascular calcification in atherosclerosis [230]. Additionally, in senescent VSMCs, tumor necrosis factor receptor-associated protein 1 (TRAP1) can promote lactate-induced H4K12la modification, which can promote the expression of senescence-associated secretory phenotype (SASP)-associated genes in VSMCs, exacerbating atherosclerosis development [231].

Other epigenetic modifications, such as methylation and acetylation, are also involved in various phenotypes of VSMCs during atherosclerosis. For example, a recent study revealed that H3R8dime and H4R3dime induced by protein arginine methyltransferase 5 (PRMT5), which was up-regulated, are associated with VSMC phenotype switching during atherosclerosis [232]. Additionally, DOT1L, which was up-regulated, can induce H3K79me2 and is associated with VSMC phenotype switching during atherosclerosis [233]. Moreover, a variety of HDACs, such as HDAC1, 4, 5, and 9 and SIRT6, also participate in the abnormal phenotypes of VSMCs in atherosclerosis [234]. To date, many articles have reported that DNA hypermethylation and hypomethylation are also involved in various abnormal phenotypes of VSMCs in atherosclerosis. We will not elaborate further in this paper; please refer to the corresponding excellent reviews for details [234–237]. Through numerous literature searches, we found that in atherosclerosis, methylation and acetylation are not involved in the abnormal phenotype by affecting their metabolism; perhaps the underlying metabolic mechanism has not been identified.

### Endothelial cells

Endothelial cells constitute the first line of defense against various harmful substances in the blood. Healthy endothelial cell structure and function are the keys to maintaining vascular homeostasis, whereas endothelial cell dysfunction is the initial factor leading to the progression of atherosclerotic plaques [238, 239]. Hypertension, hyperglycemia, hyperlipidemia, and hemodynamic disorders are important causes of structural and functional disorders in endothelial cells [237], but the specific mechanisms are not fully understood. Like macrophages and VSMCs, endothelial cells also undergo metabolic and epigenetic alterations during atherosclerosis (Fig. 5). Under normal conditions, owing to their low mitochondrial content, endothelial cells rely mainly on aerobic glycolysis for energy production [240]. The rate of glycolysis in endothelial cells is further increased in atherosclerosis [241]. An appropriate increase in the rate of glycolysis is beneficial to the proliferation of endothelial cells, which protects the vascular wall and subsequently inhibits the progression of atherosclerosis. However, excessive glycolysis can lead to abnormal proliferation of endothelial cells, stimulate angiogenesis, damage the normal structure of blood vessels, and then aggravate atherosclerosis [2]. The glycolytic metabolite lactate may play a key role in the above process. An increase in lactate levels in the body caused by exercise can induce Mecp2k271 lactylation in endothelial cells to inhibit the expression of inflammatory factors, which can promote the expression of endothelial nitric oxide synthase (Enos) and inhibit the development of atherosclerosis [242]. However, another study reported that P300/antisilencing factor 1 A (ASF1A) molecular complex-mediated H3K18la could promote the expression of Snail1 in endothelial cells, thereby promoting EndMT and aggravating atherosclerosis [243].

Additionally, studies have shown that different stimuli, such as turbulent blood flow (TBF) and ox-LDL, can also affect the epigenetic status of endothelial cells in atherosclerosis [244]. For example, under ox-LDL stimulation, DNMT3b, which was increased, can induce the hypermethylation of cellular repressor of E1A-stimulated genes (CREGs) in endothelial cells, leading to endothelial cell dysfunction [245]. Furthermore, ox-LDL can also trigger the expression of DNMT1 in endothelial cells, which can promote Kruppel-like factor 2 (KLF2) hypermethylation and aggravate inflammation [246]. Under oscillatory shear stress (OSS), the upregulation of DNMT1 and DNMT3a can mediate the hypermethylation of Kruppel-like factor 4 (KLF4), thereby inhibiting inflammation [247, 248]. In addition, OSS can also upregulate the expression of HDAC1, 2, 3, and HDAC5 and 7 in endothelial cells, which can promote endothelial cell dysfunction [234]. The above methylation, acetylation, and lactylation modifications induced by metabolites may constitute the basis of trained immunity in endothelial cells in the context of atherosclerosis.

### Conclusions and prospects

Metabolic and epigenetic alterations are common in cardiovascular diseases, and their interaction affects the development of the disease. Various metabolites produced during metabolic processes can alter the structure of chromatin by affecting epigenetic modifying enzymes in cells, thereby storing information on cellular metabolic changes in chromatin. Changes in chromatin structure may in turn affect cellular metabolism, thereby achieving a metabolism‒ epigenetic‒metabolism loop. The newly discovered lactylation modification identified in recent years is a typical example of a metabolism‒epigenetic‒metabolism loop. In Alzheimer’s disease, the rate of glycolysis increases in microglia, and the glycolytic product lactate can induce H4K12la, which in turn can drive gene transcription of glycolytic pathway enzymes, thereby achieving the glycolysis‒lactate‒H4K12la‒glycolysis loop in microglia [11]. This loop is a key factor in disease progression. Therefore, targeting these metabolic and epigenetic alterations is key to treating diseases, including cardiovascular diseases. The regulation of metabolism can be achieved by changing nutrient levels in the body, targeting key enzymes in metabolic pathways or the transport of metabolites, or alleviating hypoxia. In addition, various inhibitors of epigenetic modifying enzymes, such as HDACs, HATs, DNMTs, and KMTs, are currently available in clinical practice, but the specificity and cell targeting effects of these inhibitors are not satisfactory. Therefore, in the future, it is necessary to develop more specific epigenetic modifying enzyme inhibitors and new drug-targeted delivery carriers and explore new therapeutic functions of old drugs. Additionally, the combination of metabolism-targeted and epigenetic-targeted drugs may have profound implications in the future, but extensive basic and clinical experimental verification is needed. We believe that, in the future, metabolism- and epigenetic-targeting therapies can be effective for the treatment of cardiovascular diseases.
